# Patterns of case fatality and hospitalization duration among nearly 1 million hospitalized COVID-19 patients covered by Iran Health Insurance Organization (IHIO) over two years of pandemic: An analysis of associated factors

**DOI:** 10.1371/journal.pone.0298604

**Published:** 2024-02-23

**Authors:** Reza Mehrizi, Ali Golestani, Mohammad-Reza Malekpour, Hossein Karami, Mohammad Mahdi Nasehi, Mohammad Effatpanah, Mehdi Rezaee, Zahra Shahali, Ali Akbari Sari, Rajabali Daroudi

**Affiliations:** 1 National Center for Health Insurance Research, Tehran, Iran; 2 Non-Communicable Diseases Research Center, Endocrinology and Metabolism Population Sciences Institute, Tehran University of Medical Sciences, Tehran, Iran; 3 Pediatric Neurology Research Center, Research Institute for Children Health, Shahid Beheshti University of Medical Sciences, Tehran, Iran; 4 School of Medicine, Imam Khomeini Hospital, Tehran University of Medical Sciences, Tehran, Iran; 5 Department of Orthopedics, School of Medicine, Tehran University of Medical Sciences, Tehran, Iran; 6 Department of Health Management, Policy and Economics, School of Public Health, Tehran University of Medical Sciences, Tehran, Iran; Pasteur Institute of Iran, ISLAMIC REPUBLIC OF IRAN

## Abstract

**Background:**

Different populations and areas of the world experienced diverse COVID-19 hospitalization and mortality rates. Claims data is a systematically recorded source of hospitalized patients’ information that could be used to evaluate the disease management course and outcomes. We aimed to investigate the hospitalization and mortality patterns and associated factors in a huge sample of hospitalized patients.

**Methods:**

In this retrospective registry-based study, we utilized claim data from the Iran Health Insurance Organization (IHIO) consisting of approximately one million hospitalized patients across various hospitals in Iran over a 26-month period. All records in the hospitalization dataset with ICD-10 codes U07.1/U07.2 for clinically/laboratory confirmed COVID-19 were included. In this study, a case referred to one instance of a patient being hospitalized. If a patient experienced multiple hospitalizations within 30 days, those were aggregated into a single case. However, if hospitalizations had longer intervals, they were considered independent cases. The primary outcomes of study were general and intensive care unit (ICU) hospitalization periods and case fatality rate (CFR) at the hospital. Besides, various demographic and hospitalization-associated factors were analyzed to derive the associations with study outcomes using accelerated failure time (AFT) and logistic regression models.

**Results:**

A total number of 1 113 678 admissions with COVID-19 diagnosis were recorded by IHIO during the study period, defined as 917 198 cases, including 51.9% females and 48.1% males. The 61–70 age group had the highest number of cases for both sexes. Among defined cases, CFR was 10.36% (95% CI: 10.29–10.42). The >80 age group had the highest CFR (26.01% [95% CI: 25.75–26.27]). The median of overall hospitalization and ICU days were 4 (IQR: 3–7) and 5 (IQR: 2–8), respectively. Male patients had a significantly higher risk for mortality both generally (odds ratio (OR) = 1.36 [1.34–1.37]) and among ICU admitted patients (1.12 [1.09–1.12]). Among various insurance funds, Foreign Citizens had the highest risk of death both generally (adjusted OR = 2.06 [1.91–2.22]) and in ICU (aOR = 1.71 [1.51–1.92]). Increasing age groups was a risk of longer hospitalization, and the >80 age group had the highest risk for overall hospitalization period (median ratio = 1.52 [1.51–1.54]) and at ICU (median ratio = 1.17 [1.16–1.18]). Considering Tehran as the reference province, Sistan and Balcuchestan (aOR = 1.4 [1.32–1.48]), Alborz (aOR = 1.28 [1.22–1.35]), and Khorasan Razavi (aOR = 1.24 [1.20–1.28]) were the provinces with the highest risk of mortality in hospitalized patients.

**Conclusion:**

Hospitalization data unveiled mortality and duration associations with variables, highlighting provincial outcome disparities in Iran. Using enhanced registry systems in conjunction with other studies, empowers policymakers with evidence for optimizing resource allocation and fortifying healthcare system resilience against future health challenges.

## Introduction

The COVID-19 pandemic imposed a sudden and heavy burden on public global health with its vast impacts on different aspects of human life, with the most important one being a significant threat to life [1]. Estimations on the real figures of COVID-19 mortality and associated factors and predictors have been the focus of researchers and public health investigators in the past couple of years [2, 3]. Two years after COVID-19 spread in Iran, a country that was heavily affected by the disease, investigations on the all-cause mortality statistics revealed that the actual death toll was higher than reported, with differences in sociodemographic and geographical factors across the country [4]. Besides the infection and its complications, the associated clinical and non-clinical factors also contributed to the severity of the disease and the potential adverse outcomes like death. Among these factors, comorbidities like chronic non-communicable diseases (NCDs) and different healthcare access and utilization levels were noticeable [5–9].

Among various determinants of COVID-19 hospitalization and mortality, the demographic and clinical factors are the most investigated, with varying levels of importance and contribution [10, 11]. However, another important aspect of disease management that also affects the outcomes and contributes to hospitalization and adverse outcomes like death is health-system-related factors, including the quantity and quality of healthcare services and various challenges the healthcare systems face during a viral pandemic like COVID-19 [12]. Several previous studies in Iran have utilized data from hospitalized patients to examine the association of various demographic and clinical variables with COVID-19 outcomes [13–16]. However, these studies were constrained by their focus on single or multiple centers, diverse time frames during the pandemic, and different geographical locations within Iran. Additionally, some studies specifically investigated the impact of COVID-19 on certain populations, such as patients with cancer [17]. These limitations restrict the generalizability of COVID-19 outcomes in Iran and, more importantly, hinder the ability to compare outcomes and health-system-related factors at subnational levels.

The health claims data is a source of information on patients’ hospitalization, management, and outcomes provided by organizations like insurance companies and the health system authorities, which enable the investigators to assess the function of the health system and healthcare providers in the process of patient management on a wider scale [18, 19]. The Iran Health Insurance Organization (IHIO) is the provider of essential health insurance services and has a vast coverage of nearly 50% of the Iranian population [20]; it is also heavily involved in coverage of the healthcare costs of patients hospitalized with COVID-19 [5]. Utilizing data from organizations like IHIO could help investigate the outcomes of diseases such as COVID-19 at the national level, facilitating more rigorous comparisons among provinces and hospitals, and allowing an assessment of the performance of the health system. Previous research on the IHIO hospitalization registry data on COVID-19 for the first year of the pandemic in Iran showed the case fatality rate (CFR) of this disease among hospitalized patients was about 14.0% and higher male patients, with disparities among provinces of the country [21]. However, the infection patterns differed majorly in the following years of the pandemic in Iran as the delta and omicron variants spread among the population [22].

In this study, we aimed to investigate hospitalization registry data from IHIO, focusing on the patterns of hospitalization days and mortality among patients with COVID-19 over a two-year period across all provinces of Iran. By considering the large and diverse sample from all provinces in the IHIO database and the extended study period, the results of this study can provide a better understanding and comparison of the insurance and hospitalization associated factors in all provinces of Iran during the COVID-19 era. This information is crucial for improving the workflow of the health system in managing infectious pandemic cases and enhancing the quality of care to save more patients in probable future pandemics.

## Materials and methods

### Study design and population

In this retrospective registry-based study, we retrieved the data of hospitalized patients admitted with COVID-19 at hospitals collaborating with the IHIO in all provinces of Iran for 26 months, from February 1, 2020 to March 20, 2022. During this period, approximately 42 million residents of Iran were covered by IHIO. The entire IHIO hospitalization database was used to retrieve this data. Patients who were admitted and recorded with International Classification of Diseases 10th revision (ICD-10) codes U07.1 (indicating a COVID-19 diagnosis through laboratory tests) and U07.2 (indicating a COVID-19 diagnosis made by clinicians based on clinical signs and symptoms) in the IHIO hospitalization database were extracted, resulted in overall 1,113,678 admissions. The extracted data included information on patients’ demographic, hospitalization, and insurance details. Access to the fully anonymized data was granted to researchers in January 2023, at which point they commenced their analysis. Due to the real-world registry-based nature of this study, which included a substantial portion of patients equivalent to nearly the entire population under IHIO coverage who experienced hospitalization during the pandemic period, calculating sample size was not required and we used all available data in our analysis.

### Data source variables

In the hospitalization dataset retrieved for analysis, each admission had numerous variables. Every patient was uniquely identified by a patient code, facilitating the recognition of multiple admissions by the same individual. The dataset contained COVID-19 diagnoses, coded as U07.1 and U07.2 following the ICD-10 classification. Demographic characteristics, encompassing ages ranging from 1 to 105 years and gender (male/female), were also part of the dataset. Within the IHIO framework, there were various funds, including Rural, Civil Servants, Universal Health Insurance, Iranian, Foreign Citizens, and Other Social Strata funds. The assignment of each individual to a specific fund is determined by factors such as occupation, income, and socioeconomic status [21].

The dataset encompassed the province of hospitalization, including all provinces in Iran. The type of admission, differentiating between ward and emergency department admissions, was recorded in the dataset. This classification may be indicative of the patient’s initial condition upon hospital arrival, as more severe cases often directly enter wards when beds are available. Furthermore, the total number of hospitalization days, encompassing total days in hospitals and days spent in the intensive care unit (ICU), were available. Instances exceeding 90 days were tagged as outliers and deemed unsuitable for analysis. Additionally, the dataset included admission and discharge dates. The admission date was categorized by the month of hospitalization, a parameter that may hold relevance considering the variations in COVID-19 strains and vaccine accessibility during the pandemic, potentially influencing the severity of the disease.

Physician specializations responsible for patient care were categorized into primary groups, which included General Practitioners (GPs), infectious diseases specialists, internal medicine specialists (covering all internal specialties except pulmonologists), emergency physicians, pediatricians, cardiologists, pulmonologists, others, and unknown. This classification took into account that specialists would typically handle more severe cases, thus the specialty of the physician is a variable potentially linked to the severity of the disease. Additionally, the outcome of hospitalization was documented in the dataset, distinguishing between instances of discharge, which regraded as recovery, and death. While clinical symptoms and underlying diseases of admitted patients were crucial variables for reporting and inclusion in models, we could not include them in this study due to the lack of this data in the IHIO registry.

### Data preparation and outcomes definition

The primary step in data preparation involved defining cases for this study. In the initial data exploration, we identified instances where some patients underwent multiple hospitalizations within the study period. While it is conceivable for individuals to get COVID-19 multiple times, especially given the extended duration of our study, we observed a common pattern wherein consecutive admissions occurred within days or very short intervals. This pattern could be attributed to some hospitalizations being brief, primarily for medication administration or early discharges before full recovery. Consequently, we aggregated all hospitalizations within intervals of 30 days and less for a single patient into a unified case, representing a single entry in the curated dataset. For this newly defined case, the date of the first hospitalization was considered as the date of hospitalization. Regarding patients with only one admission, that admission was directly considered as a case. In instances where patients experienced multiple admissions with intervals surpassing one month, each hospitalization was treated as an independent case. The total number of hospitalization days in hospital and intensive care unit (ICU) was computed for multiple admissions. If a case consisting of several consecutive admissions, had a recorded instance of death, it was designated as the outcome; conversely, recovery was presumed as the hospitalization outcome for that case. Comprehensive details concerning alterations to other variables in these cases are elucidated in the S1 File.

This study had two primary outcomes. The case fatality rate (CFR), as one of the primary outcomes of the study, was defined as the proportion of cases in which the outcome of hospitalization was death in the dataset. It is noteworthy that all cases for which the final outcome of hospitalization was not death and were discharged, were considered as recovered cases. The other outcome was hospitalization days. Both outcomes were investigated in all hospitalized population and patients who experienced the ICU. This approach aimed to generalize the results across both patient groups.

### Statistical analysis

Quantitative variables were summarized by mean and standard deviation (±SD) or median and interquartile range (IQR), and categorical variables were summarized by frequency and percentage, and we reported 95% confidence interval (CI) for representing effect size and statistical significance comparison. The contribution of various associated factors to the mortality of cases was analyzed by univariable and multiple logistic regression, and results were reported in crude odds ratio (OR) and adjusted odds ratio (aOR) with a 95% confidence interval (CI). Concerning hospitalization duration, we used a parametric survival model, Accelerated Failure Time (AFT) model, in which recovered and deceased cases were regarded as events and censored. Following an assessment of various distributions, the log-logistic distribution emerged as the most fitting choice, demonstrated by its attainment of the lowest Akaike information criterion (AIC). This distribution was then utilized to model the outcome in conjunction with different variables. The findings of both univariable and multiple analyses were presented as crude median ratios (MR) and adjusted median ratios (MR), accompanied by a 95% confidence interval (CI). In adjusted models for both outcomes, age and sex were considered as variables reflecting basic characteristics of the cases. Province and insurance fund were regarded as variables contributing to the socioeconomic aspect of the cases, while admission type, month of admission, and specialty of the physician were considered variables reflecting the severity of the disease. Data was prepared using Python programming language v3.11.4 (Pandas and Numpy libraries [https://www.python.org/]). Visualizations were done by the Matplotlib library in Python and the ggplot2 library in R programming software v4.3.1 (https://cran.r-project.org/). Statistical analyses were done by Statsmodels and Lifelines libraries in Python.

### Ethics statement

This study was done according to the Declaration of Helsinki guidelines, and the study protocol was reviewed by the ethical committee at Tehran University of Medical Sciences and received ethical approval before initiation of the investigation (code: IR.TUMS.SPH.REC.1401.120). Considering this study was a retrospective study on medical records, The provided data by IHIO in this study were fully anonymized before investigators had access to it, and the requirement for informed consent was not necessary and has been waived by the ethics committee.

## Results

### General findings

A total of 1 113 678 admissions with COVID-19 diagnosis at hospitals registered with IHIO services were recorded in this study, including 649 265 (58.30% [95% CI: 58.21–58.39]) with U07.1 and 464 413 (41.70% [41.61–41.79]) with U07.2 diagnostic code during the 26-month study period. Admission types were 822 509 (73.86% [73.77–73.94]) ward admissions and 291 169 (26.14% [26.06–26.23]) emergency department admission. The top clinicians managing the admissions were general practitioners (43.63% [43.54–43.72]), internal medicine specialists (17.43% [17.36–17.50]), and infectious diseases specialists (16.82% [16.75–16.89]), while anesthesiologists handled the least of the admissions (0.47% [0.46–0.49]). The number of extracted admissions was for 876 828 unique patients, with 83.73% (95% CI: 83.63–83.79) hospitalized only once, 94.63% (94.57–94.67) hospitalized a maximum of two times, and only 5.37% (5.32–5.42) had ≥3 hospitalizations.

**Fig 1:**
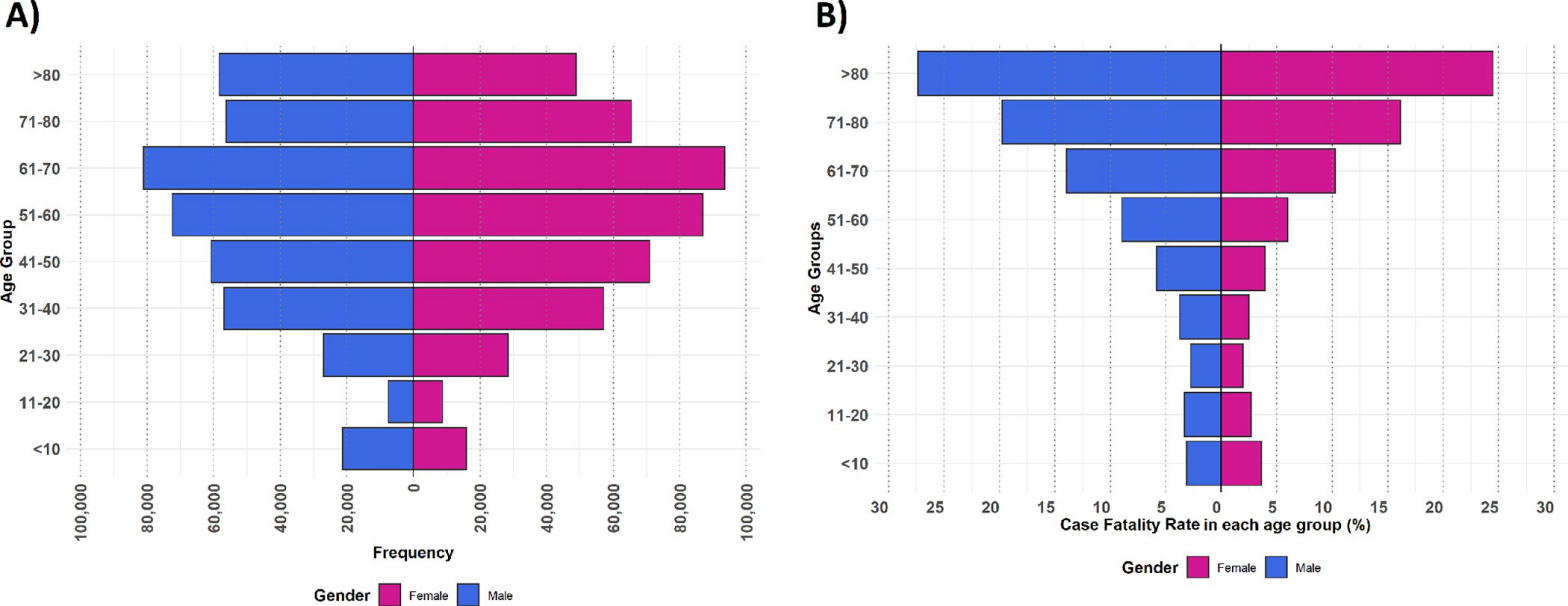
The population age pyramid. A) defined cases, B) Case Fatality Rate.

**Fig 2:**
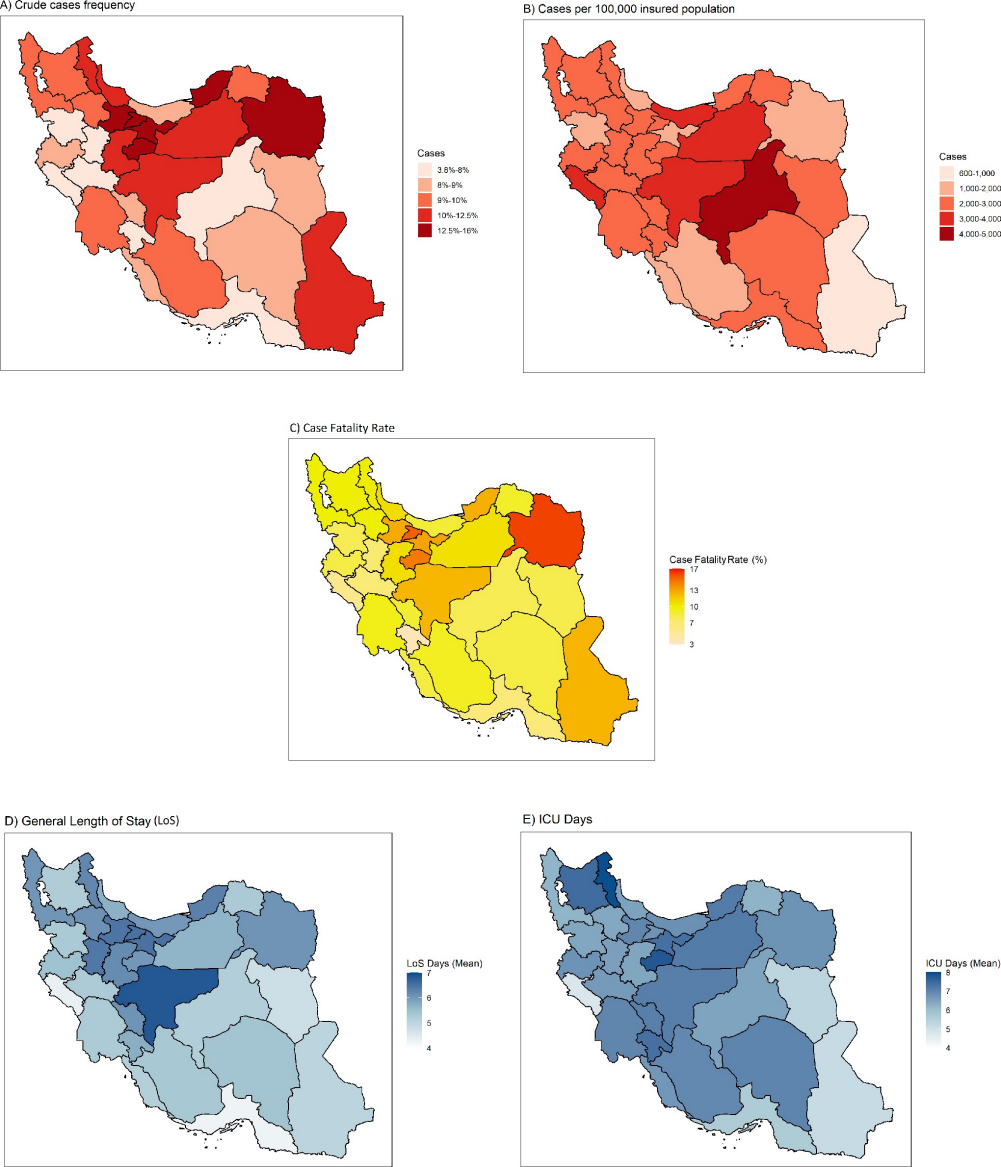
Subnational distribution of COVID-19 in provinces of Iran. A) crude number of cases, B) number of cases per 100 000 insured population, C) case fatality rates, D) general hospitalization length of stay, E) ICU hospitalization length of stay.

According to the case definition based on hospitalization periods, 917 198 cases were defined based on criteria, among which 476 140 (51.91% [95% CI: 51.81–52.01]) were females and 441 058 (48.09% [47.99–48.19]) were males (Table 1). For each sex, the 61–70 age group had the highest number of cases as 81 048 (18.37% [18.26–18.49]) males and 93 574 (19.65% [19.53–19.76]) females, and the 11–20 age group had the least number of cases as 7508 (1.70% [1.66–1.74]) males and 8814 (1.85%) females [1.82–1.88] (Fig 1A). The distribution of the crude number of cases (Fig 2A) and standardized number of cases per 100 000 insured population (Fig 2B) in each province had variations across the country. Total number of cases was highest in Khorasan, Razavi (71558) and least in Bushehr (8427), while based on standardized values (per 100 000 insured population), Yazd (4128) province had the highest value and Sistan and Baluchestan (637) had the least value (S1 Table). Among provinces, the 51–60 and 61–70 age groups had the highest frequency of cases in almost all provinces (S1 Fig). Most cases were visited by general practitioners (362 261 cases, constituting 39.50% [39.40–39.60] of all cases), while the fewest visited cases, 3269 (0.36% [0.34–0.37]), were related to anesthesiologists (Table 1).

**Table 1:** Case fatality and recovery rates based on different stratifications among included cases.

Stratification	Outcome, No. (%) (95% Confidence Interval)		
	Recovered	Dead	Total
**Sex**			
Male	388924 (88.18%) (88.08–88.28)	52134 (11.82%) (11.72–11.92)	441058 (48.09%) (47.99–48.19)
Female	433296 (91.0%) (90.92–91.08)	42844 (9.0%) (8.92–9.08)	476140 (51.91%) (51.81–52.01)
**Age group**			
<10	35800 (96.65%) (96.47–96.84)	1239 (3.35%) (3.16–3.53)	37039 (4.04%) (4.00–4.08)
11–20	15836 (97.02%) (96.76–97.28)	486 (2.98%) (2.72–3.24)	16322 (1.78%)(1.75–1.81)
21–30	53976 (97.67%) (97.54–97.79)	1289 (2.33%) (2.21–2.46)	55265 (6.03%) (5.98–6.07)
31–40	110470 (96.89%) (96.78–96.99)	3551 (3.11%) (3.01–3.22)	114021 (12.43%) (12.36–12.50)
41–50	125454 (95.21%) (95.10–95.33)	6308 (4.79%) (4.67–4.90)	131762 (14.37%) (14.29–14.44)
51–60	147637 (92.68%) (92.55–92.81)	11658 (7.32%) (7.19–7.45)	159295 (17.37%) (17.29–17.45)
61–70	153718 (88.03%) (87.88–88.18)	20904 (11.97%) (11.82–12.12)	174622 (19.04%) (18.96–19.12)
71–80	100061 (82.19%) (81.98–82.41)	21677 (17.81%) (17.59–18.02)	121738 (13.27%) (13.20–13.34)
>80	79268 (73.99%) (73.73–74.25)	27866 (26.01%) (25.75–26.27)	107134 (11.68%) (11.61–11.75)
**Specialty**			
Anesthesiologist	1651 (50.5%) (48.79–52.22)	1618 (49.5%) (47.78–51.21)	3269 (0.36%) (0.34–0.37)
Cardiology	8817 (86.73%) (86.07–87.39)	1349 (13.27%) (12.61–13.93)	10166 (1.11%) (1.09–1.13)
Emergency	34755 (90.41%) (90.12–90.71)	3686 (9.59%) (9.29–9.88)	38441 (4.19%) (4.15–4.23)
General practitioner	329175 (90.87%) (90.77–90.96)	33086 (9.13%) (9.04–9.23)	362261 (39.50%) (39.40–39.60)
Infectious Disease	140754 (90.37%) (90.23–90.52)	14994 (9.63%) (9.48–9.77)	155748 (16.98%) (16.90–17.06)
Internal	135578 (87.91%) (87.74–88.07)	1865 (12.09%) (11.93–12.26)	154230 (16.82%) (16.74–16.89)
Others	18086 (91.04%) (90.64–91.44)	1780 (8.96%) (8.56–9.36)	19866 (2.17%) (2.14–2.20)
Pediatrics	24336 (95.92%) (95.67–96.16)	1036 (4.08%) (3.84–4.33)	25372 (2.77%) (2.73–2.80)
Pulmonologist	7074 (77.03%) (76.16–77.89)	2110 (22.97%) (22.11–23.84)	9184 (1.0%) (0.98–1.02)
Unknown	121994 (87.98%) (87.81–88.15)	16667 (12.02%) (11.85–12.19)	138661 (15.12%) (15.04–15.19)
**Insurance funds**			
Rural	307688 (90.80%) (90.70–90.90)	31180 (9.2%) (9.10–9.30)	338868 (36.95%) (36.85–37.04)
Civil Servants	202857 (88.69%) (88.56–88.82)	25857 (11.31%) (11.18–11.44)	228714 (24.94%) (24.85–25.02)
Other Social Strata	85709 (85.49%) (85.27–85.71)	14544 (14.51%) (14.29–14.73)	100253 (10.93%) (10.87–10.99)
Foreign Citizens	4448 (81.87%) (80.85–82.89)	985 (18.13%) (17.11–19.15)	5433 (0.59%) (0.58–0.61)
Iranian	28837 (85.01%) (84.63–85.39)	5086 (14.99%) (14.61–15.37)	33923 (3.7%) (3.66–3.74)
Universal Health Insurance	190984 (91.79%) (91.68–91.91)	17074 (8.21%) (8.09–8.32)	208058 (22.68%) (22.60–22.77)
Unknown	1697 (87.07%) (85.58–88.56)	252 (12.93%) (11.44–14.42)	1949 (0.21%) (0.20–0.22)
**Admission type**			
Ward admission	663686 (87.82%) (87.75–87.90)	92011 (12.18%) (12.10–12.25)	755697 (82.39%) (82.31–82.47)
Emergency department	158534 (98.16%) (98.10–98.23)	2967 (1.84%) (1.77–1.90)	161501 (17.61%) (17.53–17.69)
**ICU admission**			
Negative	630634 (95.1%) (95.04–95.15)	32527 (4.9%) (4.85–4.96)	663161 (81.74%) (81.65–81.82)
Positive	88347 (59.63%) (59.38–59.87)	59824 (40.37%) (40.13–40.62)	148171 (18.26%) (18.18–18.35)
**Total**	822220 (89.64%) (89.58–89.71)	94978 (10.36%) (10.29–10.42)	917198

### COVID-19 mortality and associated factors

**Fig 3:**
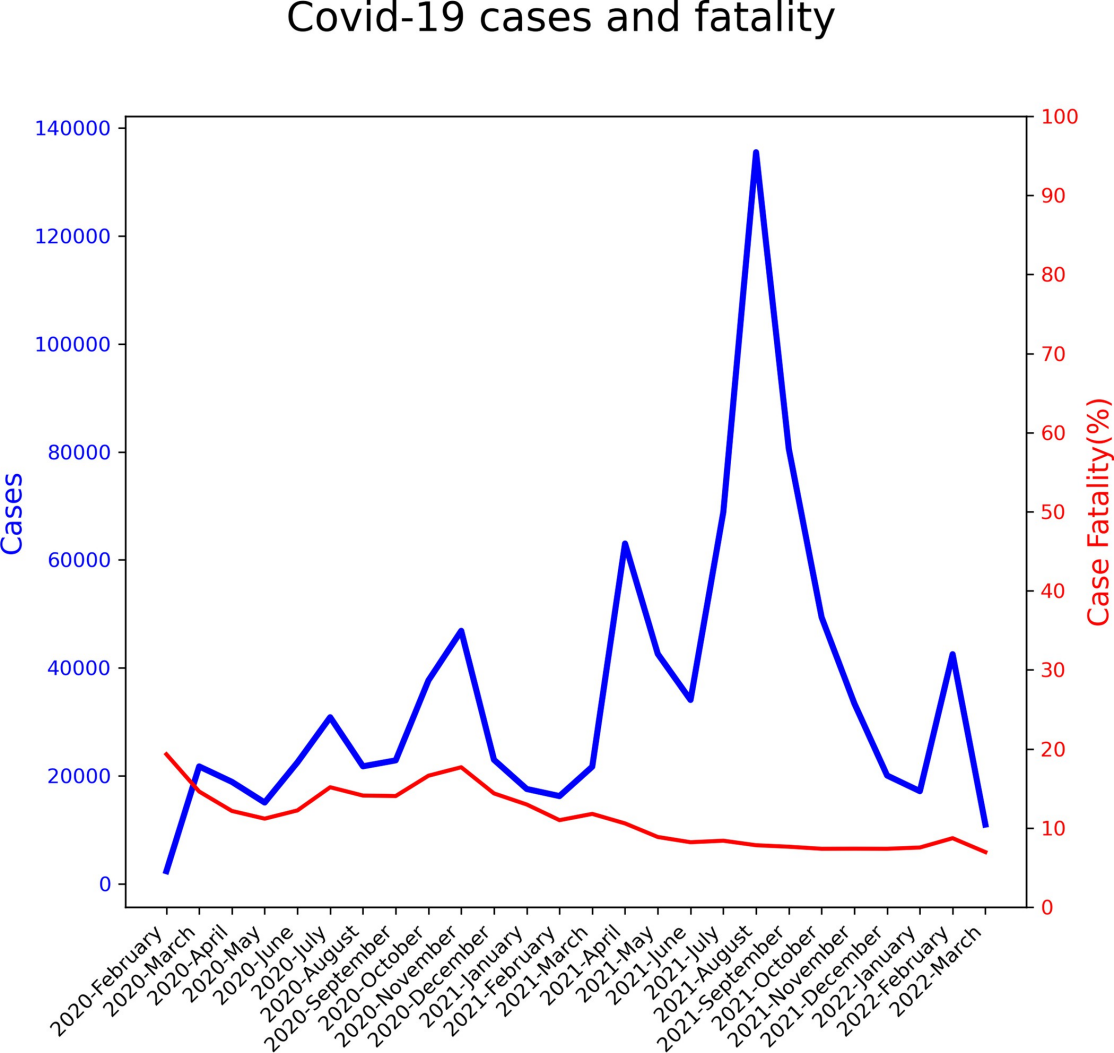
COVID-19 cases and fatalities trends throughout the study period.

Among the 917 198 defined cases, 94 978 deaths and 822 220 recoveries were recorded, resulting in a CFR of 10.36% (95% CI: 10.29–10.42). Considering total cases of each sex, CFR was 11.82% (11.72–11.92) among males and 9.00% (8.92–9.08) among females which were different significantly. Regarding total cases in each age group, the 21–30 group had the statistically significant lowest CFR (2.33% [95%CI: 2.21–2.46]), while the >80 age group had the statistically significant highest value (26.01% [25.75–26.27]) (Table 1). However, the pattern of CFR among different age groups in both sexes was almost similar (Fig 1B). Across the country, Khorasan Razavi (16.11% [15.84–16.38]) had the highest CFR, followed by Alborz (15.35% [14.82–15.89]) and Qom (14.55% [14.02–15.08]), while Kohgiluyeh and Boyer-Ahmad (3.78% [3.45–4.11]) had the least CFR (Fig 2C, S2 Table). Among the total cases visited by each specialty, anesthesiologists had the significantly highest CFR (49.5% [47.78–51.21]), followed by pulmonologists (22.97% [22.11–23.84]); on the other hand, pediatricians had the significantly highest recovery rates (95.92% [95.67–96.16]). Regarding the total cases within each insurance fund, the highest CFR was significantly observed in Foreign Citizens (18.13% [17.11–19.15]), while they accounted for the smallest number of cases in the study (5433 cases, constituting 0.59% [0.58–0.61] of all cases). Regarding total cases of each admission type, CFR was significantly higher among ward-admitted patients (12.18% [12.10–12.25]) than emergency department-admitted patients (1.84% [1.77–1.90]). Also, cases who had the experience of ICU admission had a significant higher CFR (40.73% [40.13–40.62]) compared to others (4.9% [4.85–4.96]) (Table 1). In contrast to several peaks in case incidence during the study period, the proportion of mortalities steadily decreases over time (Fig 3). Also, the older age groups had the greatest share of dead patients in almost all sections of the study period, with minor variations in some periods (S2 Fig).

Analysis of data through statistical tests showed male patients had a significantly higher risk for mortality both generally (OR = 1.36 [95% CI:1.34–1.37]) and among ICU admitted patients (OR = 1.12 [1.09–1.12]). Older patients were remarkably at higher risk of death as patients aged>80 years had higher odds both generally (OR = 10.16 [9.58–10.77]) and in ICU admitted patients (OR = 7.08 [6.6–7.6]). The number of hospitalization days slightly increased the risk of death (aOR = 1.05 [1.05–1.06]), while the number of hospitalization days in the ICU more noticeably increased this risk (aOR = 1.18 [1.18–1.18]). Also, being admitted to the ICU increased mortality risk significantly (aOR = 13.25 [13.03–13.47]). Among various insurance funds, Foreign Citizens had the highest risk of death both generally (aOR = 2.06 [1.91–2.22]) and in ICU (aOR = 1.71 [1.51–1.92]). Compared to general practitioners, among all admitted patients, those managed with cardiologists (aOR = 0.9 [0.84–0.95]) and infectious diseases specialists (aOR = 0.85 [0.83–0.87]) had lower risk of death, while highest risk of mortality was observed anesthesiologists (aOR = 7.58 [7.01–8.2]) and pulmonologists (aOR = 2.42 [2.29–2.55]), with some variations among ICU admitted patients (Table 2).

**Table 2:** Risk of mortality due to COVID-19 based on various demographic and hospitalization-associated factors in this study.

Variables	Total cases		ICU admitted cases	
	OR	Adjusted OR	OR	Adjusted OR
**Sex**				
Female (ref)	-	-	-	-
Male	1.36 (1.34–1.37)	-	1.12 (1.09–1.14)	-
**Age groups**				
<10 (ref)	-	-	-	-
11–20	0.89 (0.8–0.99)	-	1.25 (1.1–1.43)	-
21–30	0.69 (0.64–0.75)	-	1.46 (1.32–1.61)	-
31–40	0.93 (0.87–0.99)	-	1.84 (1.7–1.99)	-
41–50	1.45 (1.37–1.55)	-	2.64 (2.45–2.85)	-
51–60	2.28 (2.15–2.42)	-	3.26 (3.04–3.51)	-
61–70	3.93 (3.71–4.17)	-	4.33 (4.04–4.65)	-
71–80	6.26 (5.9–6.64)	-	5.49 (5.11–5.89)	-
>80	10.16 (9.58–10.77)	-	7.08 (6.6–7.6)	-
**Period of hospitalization, days** *				
Overall	1.07 (1.07–1.07)	1.05 (1.05–1.06)	1.01 (1.01–1.01)	1.01 (1.01–1.01)
ICU	1.19 (1.19–1.2)	1.18 (1.18–1.18)	1.04 (1.03–1.04)	1.04 (1.04–1.04)
Positive ICU** **admission**	13.13 (12.93–13.33)	13.25 (13.03–13.47)	-	-
**Insurance type** **				
Rural (ref)	-	-	-	-
Civil Servants	1.26 (1.24–1.28)	0.99 (0.97–1.01)	1.33 (1.3–1.37)	1.08 (1.04–1.11)
Other Social Strata	1.67 (1.64–1.71)	1.26 (1.23–1.29)	1.37 (1.32–1.42)	1.16 (1.12–1.2)
Foreign Citizens	2.19 (2.04–2.34)	2.06 (1.91–2.22)	1.6 (1.43–1.8)	1.71 (1.51–1.92)
Iranian	1.74 (1.69–1.8)	1.49 (1.44–1.55)	1.24 (1.18–1.3)	1.26 (1.19–1.33)
Universal Health Insurance	0.88 (0.87–0.9)	1.19 (1.17–1.22)	0.94 (0.91–0.97)	1.13 (1.1–1.17)
Unknown	1.47 (1.28–1.67)	1.43 (1.24–1.66)	1.01 (1.01–1.01)	1.01 (1.01–1.01)
**Specialist** *				
General practitioner (ref)	-	-	-	-
Cardiology	1.55 (1.46–1.64)	0.9 (0.84–0.95)	0.31 (0.29–0.33)	0.29 (0.27–0.31)
Emergency	1.11 (1.07–1.15)	1.35 (1.3–1.41)	1.75 (1.62–1.9)	1.62 (1.49–1.77)
Infectious Disease	1.06 (1.03–1.08)	0.85 (0.83–0.87)	1.29 (1.25–1.33)	1.27 (1.23–1.32)
Internal Medicine	1.38 (1.35–1.41)	1.09 (1.06–1.11)	1.31 (1.27–1.36)	1.26 (1.22–1.31)
Others	0.99 (0.94–1.04)	1.13 (1.07–1.19)	0.59 (0.56–0.63)	0.73 (0.68–0.78)
Pediatrics	0.42 (0.39–0.45)	0.95 (0.88–1.04)	0.28 (0.25–0.3)	0.63 (0.57–0.69)
Unknown	1.32 (1.3–1.35)	1.3 (1.28–1.33)	1.18 (1.14–1.21)	1.15 (1.12–1.19)
Anesthesiologist	10.86 (10.11–11.66)	7.58 (7.01–8.2)	2.23 (2.06–2.42)	2.43 (2.23–2.65)
Pulmonologist	3.05 (2.9–3.21)	2.42 (2.29–2.55)	1.45 (1.36–1.56)	1.58 (1.47–1.7)

*Adjustments were done with sex, age, month of admission, province, insurance fund, and admission type.

**Adjustments were done with sex, age, month of admission, province.

**Fig 4:**
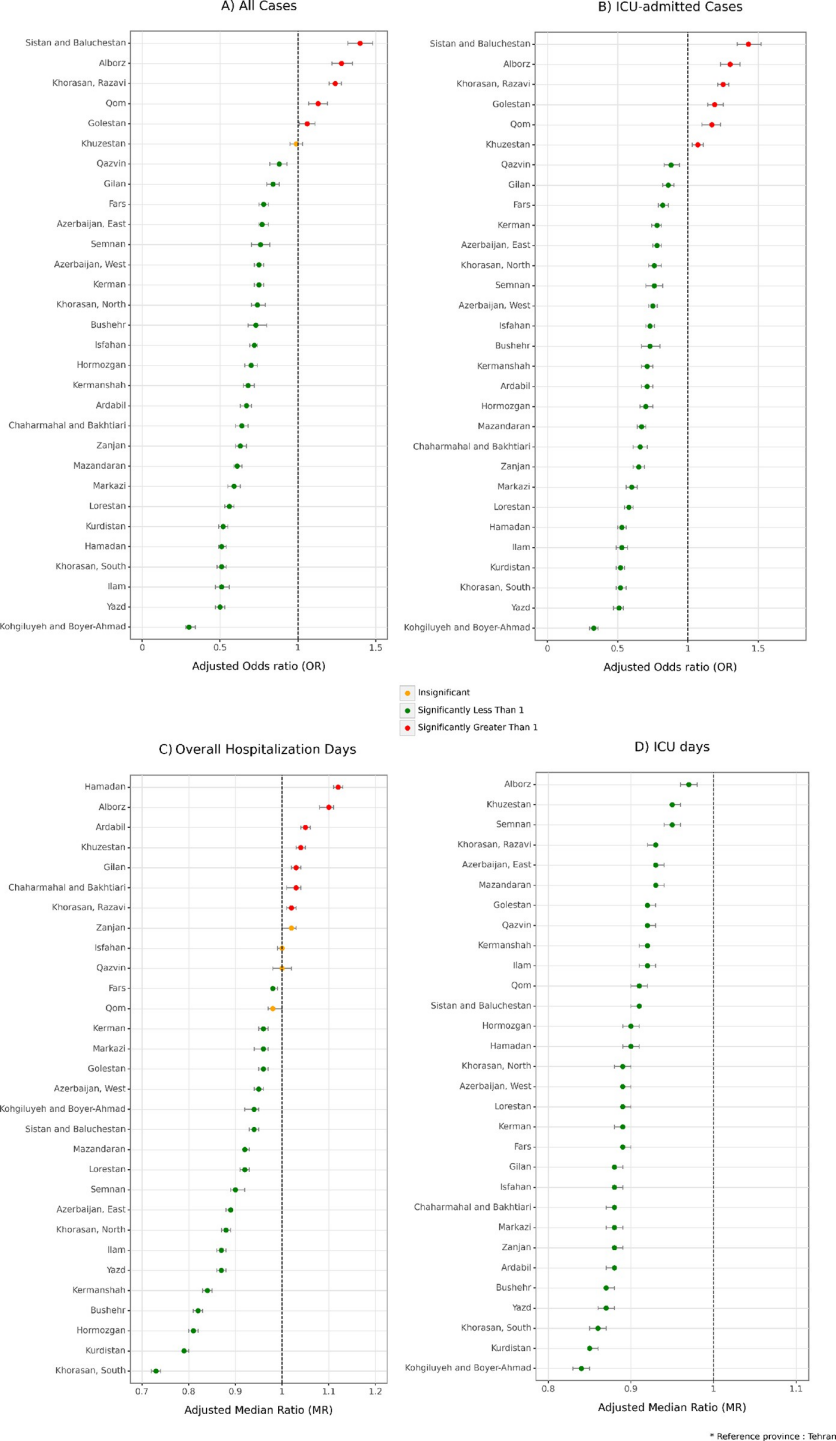
Association between provinces with hospitalization duration and mortality. A) All cases mortality, B) ICU-admitted cases, C) Overall hospitalization duration days, D) ICU duration days. (Adjustments were made with sex, age, insurance fund, admission type, the month of admission, and specialty of the physician. Tehran was considered as reference province).

In the comparison of Iranian provinces, Tehran, the capital of Iran, was considered the reference province in a model that adjusted for all possible confounding variables in the study, including sex, age, insurance fund, admission type, month of admission, and specialty. While the majority of provinces were associated with a lower risk of mortality compared to Tehran in both all admitted cases and ICU admitted cases, Sistan and Baluchestan (all hospitalized cases: aOR = 1.4 [1.32–1.48], ICU cases: aOR = 1.43 [1.35–1.52]), Alborz (all hospitalized cases: aOR = 1.28 [1.22–1.35], ICU cases: aOR = 1.3 [1.23–1.37]), and Khorasan Razavi (all hospitalized cases: aOR = 1.24 [1.20–1.28], ICU cases: aOR = 1.25 [1.21–1.29]) were associated with a higher risk of mortality in hospitalized patients (Fig 4A and 4B, S3 Table).

### Hospitalization period and associated factors

**Fig 5:**
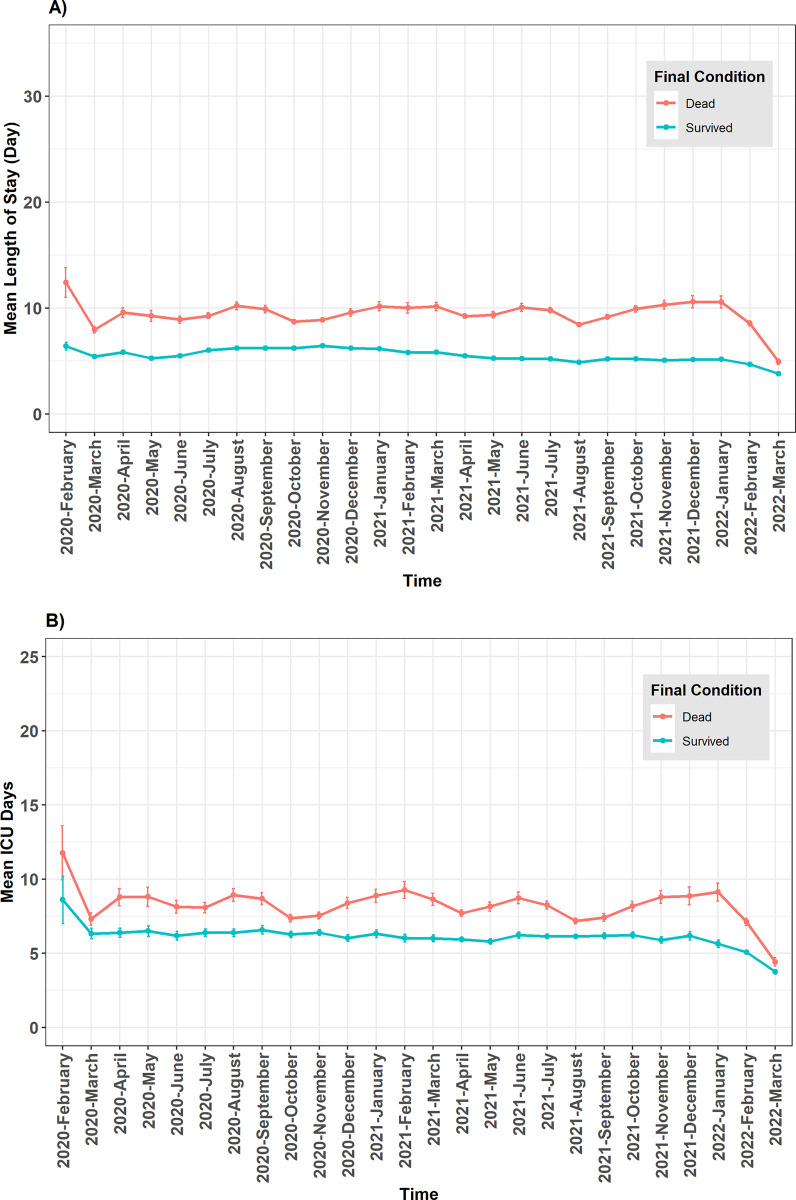
Temporal trends of mean hospitalization duration days in the study period. A) Overall hospitalization duration days, B) ICU duration days.

The overall duration of hospitalization days (mean (95%CI): 5.78 (5.77–5.80), median (IQR): 4 (3–7)) was significantly less than the duration of ICU hospitalization days (mean (95%CI): 6.85 (6.81–6.89), median (IQR): 5 (2–8)). The overall mean hospitalization period was higher among males than females but varied based on the age groups. However, the mean ICU days did not significantly differ between males and females across most age groups (S3 Fig). As it is presented in S4 Table, among insurance funds, patients with Rural insurance had the shortest hospitalization (mean = 5.53 [5.51–5.55]), and those with Iranian insurance had the longest mean hospitalization (6.78 [6.70–6.86]). The mean hospitalization period was significantly shorter at the emergency department (2.37 [2.35–2.38]) compared to the ward admission (6.51 [6.5–6.53]). Patients with recovery outcomes (9.26 [9.20–9.32]) had a hospitalization period of almost twice that of those who died (5.38 [5.37–5.39]). Patients managed by pulmonologists, anesthesiologists, and pediatricians were hospitalized for longer periods, generally and at the ICU (S4 Fig). Except for the first couple of months, the mean hospitalization days had a stable pattern during the study period and dropped in the last three months of investigation (Fig 5). Also, subnational variations in mean hospitalization were evident between provinces across the country (Fig 2D and 2E).

Survival analysis of the mean hospitalization period showed male patients had statistically significant longer hospital stay both generally (MR = 1.03 [1.03–1.04]) and in ICU (MR = 1.03 [1.03–1.03]). Increasing age groups was a risk of longer hospitalization, and the >80 age group had the highest risk for overall hospitalization period (MR = 1.52 [1.51–1.54]) and at ICU (MR = 1.17 [1.16–1.18]). Patients with ICU admission history had a remarkably longer hospital stay (aMR = 4.27 [4.24–4.30]). Also, ward admission was associated with longer hospitalization compared to the emergency department (aMR = 2.58 [2.57–2.60]). The insurance fund of Iranian was associated with a higher mean of hospitalization period compared to the Rural fund as the reference (aMR = 1.14 [1.13–1.15]). Patients managed by cardiologists (aMR = 0.82 [0.82–0.83]) and pulmonologists (aMR = 0.82 [0.82–0.83]) were associated with shorter hospitalization compared to general practitioners, while anesthesiologists were the specialty with the greatest mean of hospitalization period (aMR = 1.53 [1.51–1.56]) (Table 3).

**Table 3:** Associations between median hospitalization period and various demographic and hospitalization-associated factors.

Variables	Overall hospitalization day		ICU hospitalization day	
	MR	Adjusted MR	MR	Adjusted MR
**Sex**				
Female (ref)	-	-	-	-
Male	1.03 (1.03–1.04)	-	1.03 (1.03–1.03)	-
**Age groups**				
<10 (ref)	-	-	-	-
11–20	0.82 (0.81–0.84)	-	0.96 (0.95–0.97)	-
21–30	0.87 (0.86–0.88)	-	0.94 (0.94–0.95)	-
31–40	1.03 (1.02–1.04)	-	0.95 (0.95–0.96)	-
41–50	1.13 (1.12–1.14)	-	0.96 (0.96–0.97)	-
51–60	1.23 (1.22–1.24)	-	0.99 (0.98–0.99)	-
61–70	1.35 (1.34–1.36)	-	1.03 (1.03–1.04)	-
71–80	1.45 (1.43–1.46)	-	1.09 (1.09–1.1)	-
>80	1.52 (1.51–1.54)	-	1.17 (1.16–1.18)	-
**Positive ICU admission** *	4.61 (4.61–4.62)	4.27 (4.24–4.3)	-	-
**Admission type** *				
Emergency department (ref)	-	-	-	-
Ward	2.59 (2.58–2.6)	2.58 (2.57–2.6)	1.15 (1.15–1.15)	1.16 (1.15–1.16)
**Insurance type** *				
Rural (ref)	-	-	-	-
Civil Servants	1.1 (1.09–1.1)	1.01 (1.01–1.02)	1.03 (1.03–1.03)	1.01 (1.0–1.01)
Other Social Strata	1.16 (1.15–1.17)	1.07 (1.06–1.08)	1.05 (1.04–1.05)	1.02 (1.02–1.02)
Foreign Citizens	1.05 (1.02–1.07)	1.0 (0.98–1.03)	1.09 (1.07–1.1)	1.08 (1.06–1.09)
Iranian	1.22 (1.2–1.23)	1.14 (1.13–1.15)	1.08 (1.08–1.09)	1.06 (1.06–1.07)
Universal Health Insurance	0.99 (0.99–1.0)	1.03 (1.03–1.04)	1.0 (1.0–1.0)	1.01 (1.01–1.02)
Unknown	1.71 (1.65–1.78)	1.68 (1.62–1.75)	1.14 (1.11–1.17)	1.12 (1.09–1.15)
**Specialist** **				
General practitioner (ref)	-	-	-	-
Cardiology	1.26 (1.23–1.28)	0.82 (0.82–0.83)	1.87 (1.85–1.9)	1.75 (1.72–1.77)
Emergency	0.58 (0.57–0.59)	1.15 (1.14–1.15)	0.98 (0.97–0.98)	1.0 (0.99–1.0)
Infectious Disease	1.33 (1.32–1.34)	1.15 (1.15–1.16)	1.0 (1.0–1.0)	0.97 (0.97–0.98)
Internal Medicine	1.32 (1.31–1.33)	1.25 (1.24–1.27)	1.03 (1.03–1.04)	1.0 (1.0–1.01)
Others	1.37 (1.35–1.39)	1.22 (1.21–1.24)	1.1 (1.09–1.1)	1.09 (1.08–1.1)
Pediatrics	1.08 (1.07–1.09)	1.52 (1.52–1.53)	1.04 (1.04–1.05)	1.06 (1.05–1.07)
Unknown	1.45 (1.44–1.45)	2.34 (2.27–2.42)	1.04 (1.04–1.05)	1.06 (1.06–1.06)
Anesthesiologist	3.05 (2.94–3.16)	1.53 (1.51–1.56)	6.51 (6.32–6.69)	5.85 (5.69–6.02)
Pulmonologist	1.73 (1.7–1.77)	0.82 (0.82–0.83)	1.53 (1.5–1.55)	1.45 (1.43–1.47)

*Adjustments were done with sex, age, month of admission, province.

**Adjustments were done with sex, age, month of admission, province, insurance fund, and admission type.

In the comparative analysis of Iranian provinces, Tehran, the capital of Iran, served as the reference province in a model that accounted for all potential confounding variables in the study, including sex, age, insurance fund, admission type, month of admission, and specialty. All provinces were associated with shorter hospitalization periods in the ICU compared to Tehran, with Kohgiluyeh and Boyer-Ahmad exhibiting the lowest duration (aMR = 0.84 [0.83–0.85]). However, the pattern in total hospitalization days varied, with patients in Hamdan and Alborz at highest risk of prolonged hospital stays, with aMRs of 1.12 (1.11–1.13) and 1.10 (1.08–1.11), respectively. In contrast, Zanjan, Isfahan, Qazvin, and Qom did not show a significant difference compared to Tehran, and South Khorasan was associated with the shoertest hospital days with aMR of 0.73 (0.72–0.74) (Fig 4C and 4D, S3 Table).

## Discussion

This study investigated the hospitalization patterns due to COVID-19 in hospitals accepting patients with IHIO insurance. It revealed the various patient- and hospitalization-associated factors contributing to the mortality and hospitalization duration for the included sample of about one million Iranians. The main findings of this study were that one in ten hospitalized patients with diagnosis of COVID-19 died during the study period, with a higher prevalence among men and the elderly. Factors such as insurance fund, the managing specialist, and ICU admission during hospitalization were associated with both the length of stay and mortality risk. The results from adjusted models indicated variations in both mortality and duration of hospitalization among provinces, with a higher risk of both in provinces such as Tehran, Alborz, and Khorasan Razavi.

The most prominent finding of this study was the CFR of 10.48% among patients hospitalized with COVID-19 in Iran, which was comparable to similar publications. In a similar study on the patients with hospitalization records of COVID-19 who were also insured with the IHIO, the CFR was 14.0% [21]. In another study from a referral hospital in Tehran, the capital of Iran, the COVID-19 CFR was reported as 10.8% [23]. Another multi-center study from Tehran on more than 16000 hospitalized patients found a 10.5% value for this outcome [24]. Reports of studies from other countries had a wide range, and some of them reported 20–30% CFR for COVID-19 [25–27]; however, the in-hospital COVID-19 CFR in hospitals in the United States was in the 9–15.6% range [28], which was more in consistency with our findings. There could be several justifications for the variations in COVID-19 mortality rates in different studies and locations of investigation. The most noticeable factor responsible for this variation could be differences in populations’ characteristics and distinct patterns of risk factors in different countries [29]. Variations in study designs, periods of study, criteria for inclusion of population, and analysis variations also partly contribute to the differences in COVID-19 mortality rates.

In our study, the median and IQR of overall hospitalization days were 4 and 3–7, respectively, which aligns with similar investigations. A study in Italy reported a median Length of Stay (LoS) of 6 [30]. A systematic review comparing LoS in 46 Chinese studies with eight studies from the USA, UK, and Europe found that in China, LoS ranged from 4 to 53, while in other countries, it ranged from 4 to 21 [31]. The summary distributions estimated a median LoS of 14 (IQR: 10–19) in China and 5 (IQR: 10–19) in other countries. These variations may be attributed to differences in populations, as well as diverse policies and strategies for controlling and treating COVID-19. Importantly, getting vaccinated is proven to be linked with a shorter LoS, and this could be a reason for the variations [32, 33]. Thus, the observed changes in LoS could serve as a proxy for assessing the effectiveness of strategies implemented by policymakers. Given the absence of a significant reduction in hospitalization days throughout the two-year study of the COVID-19 pandemic in Iran until the limited last months of the study, it suggests that the strategies employed may not have been entirely successful in controlling the disease’s hospitalization outcomes, at least among IHIO-covered patients.

Increasing COVID-19 mortality and duration of hospitalization with aging in admitted patients were the other notable findings in this study. Among the population included in the current investigation, about 44% were older than 60 years old, indicating a higher probability of hospitalization and severe infection in the elderly. This finding was consistent with evidence that shows mortality due to COVID-19 increases with age, and most deaths due to this viral respiratory infection happen in the older population [34]. Although one study from Iran reported the highest COVID-19 incidence in mid age ranges of 25–64 years, the population aged older than 64 years had significantly higher mortality rates [24]. Other studies also found that patients older than 60 had mortality odds five times higher than the younger population [35], and the odds of death at an age higher than 50 were 15.4 times higher than ages below 50 [36]. Furthermore, other studies have shown that older ages were associated with a higher hospitalization duration [30, 37, 38]. This trend could have been due to increasing comorbidities with aging predisposing the individuals to a higher chance of COVID-19 incidence, its severe states, and adverse outcomes like death [4, 19]. Among comorbidities, NCDs like cardiovascular diseases and diabetes comprise the most significant proportion associated with severe COVID-19 and outcomes like death based on literature [7, 9, 39]. Therefore, careful care of older patients in both aspects of infection prevention and disease course management is needed to save more frail population.

In this study, male patients were at higher risk of COVID-19 mortality in overall hospitalized and ICU-admitted patients. The results on patterns of diseases between the two sexes were almost similar to previous studies. In a systematic review and meta-analysis of the literature, male sex had 1.86 higher odds of mortality due to COVID-19 [36]. Studies from different regions of the world show a lower incidence and adverse outcomes of COVID-19 among females and better disease prognosis in the short- and long-term [40, 41]. Similar viral respiratory epidemics like the severe acute respiratory syndrome (SARS) and the Middle East respiratory syndrome (MERS) also had similar sex involvement patterns [42, 43]. Differences between the two sexes, like genetic factors, immune-related determinants, sex hormones contribution to disease course, and behavioral variations, are among the justifications for this finding [41, 44, 45]. Thus, male patients need further precise care in the management of COVID-19.

One important feature of the current study was the investigation of the study outcomes based on the IHIO insurance fund. As revealed, the foreigner and refugee populations were at significantly higher mortality risk due to COVID-19. Refugees comprise a noticeable proportion of the foreign population living in Iran, especially those originating from Afghanistan, and their healthcare services needs put a major challenge before the Iran healthcare system and workers both during the COVID-19 pandemic and generally [21, 46]. Reports show that during the recent pandemic in Iran, approximately 124000 Afghan refugees registered for the IHIO health insurance coverage. Unfortunately, many others without this coverage cannot afford healthcare services adequately, leading to higher rates of severe infection and mortality in Iran [46]. Late presentation due to financial shortages and lack of health insurance is also noted among refugees in other countries and not only among refugees but also among those residents with minor ethnic origins like a study from the United Kingdom that reported a higher excess risk of COVID-19 contamination and adverse outcomes among minority ethnic populations [47]. Besides the low- and middle-income host countries like Iran, it is also shown that migrants in high-income countries are at higher risk of COVID-19 incidence and mortality due to various inherent characteristics and access to healthcare services and utilization [48]. Further research in health services coverage and utilization among refugees is needed to highlight the burden of diseases among this vulnerable population [49].

In this study, being admitted to ICU was dramatically associated with longer hospitalization and higher mortality rates. The odds of mortality among those needing ICU care were about 13 times that of other hospitalized patients with COVID-19. A similar publication on the IHIO database for a shorter period of data collection found this risk 7.5 times higher [21]. Other studies on hospitalized cases in Tehran showed a 3- and 3.1-times higher risk of mortality in ICU-admitted patients [14, 24]. Similar patterns and numbers were observed in studies related to other provinces of Iran, such as 5.12 and 4.35 [15, 50]. These differences could be attributed to different COVID-19 variants being prevalent in different waves, and the study periods varying among these studies. The delta variant, for example, had noticeably higher rates of severe infection and mortality in Iran [51]. The CFR of ICU-admitted cases in our study was approximately 40%. This result in consistent with a systematic review and meta-analysis of studies, which found a pooled CFR in ICU-admitted patients as high as 41.6%, ranging from 0 to 84% [52], and 48.7% in another study [53]. ICU-admitted COVID-19 patients had higher mortality rates compared to other respiratory conditions and viral pneumonia. This could be because COVID-19 has a specific disease course and spread quickly during the recent pandemic, causing a shortage of crucial ICU resources like ventilators in many places [6, 52]. Furthermore, similar to our study, other studies have shown an increased risk of hospitalization associated with ICU admission [30, 54]. This observation is attributed to the admission of severe symptomatic patients to the ICU, who may have more underlying diseases and require more complex and additional treatment and care, leading to a longer hospitalization duration.

Drawing patterns of hospitalization period and mortality rates based on the specialty of clinicians managing the hospitalized patients was the other strength of the current investigation. Due to serious healthcare worker shortages during the COVID-19 pandemic, almost all specialties had to care for patients with COVID-19 in different wards [55, 56]. However, the analyzed data in this study showed specialties like pulmonologists and anesthesiologists had higher rates of patient loss, which is justifiable as those with severe infection and poorer prognosis are more managed by these specialists. Considering this notion in evaluating the patient management process by different specialties is essential to avoid biased inference and interpret the data appropriately. Notably, the study showed that managing patients by cardiologists and infectious disease specialists were associated with lower mortality compared to general practitioners (GPs). Therefore, further studies using causal inference techniques are needed to determine if, in scenarios like pandemics, it is rational to include all specialties in the management of patients. This approach is similar to what Farzadfar et al. did to demonstrate the efficacy of using health workers (Behvarzs) in the management of hypertension and diabetes in primary healthcare in Iran [57].

Our study revealed disparities in mortality and hospitalization duration among provinces, aligning with geographical variations observed in both previous Iranian and other countries studies [21, 58, 59]. In comparison to a similar study on the IHIO database covering an one year shorter period [21], Sistan and Baluchestan, Khorasan Razavi, Qom, and Golestan consistently showed higher mortality risks. However, our study uniquely identified Alborz as a province associated with elevated mortality and hospitalization duration. The variations among geographical locations may be attributed to diverse factors, including demographic characteristics [60], differences in insurance funds [21], and the epidemiology and severity of the disease [61, 62]. Our models were adjusted for demographic characteristics such as age and sex, as well as other factors like insurance fund, admission type, month of admission, and physician specialty. This suggests that the comparison of provinces in our models could be influenced by other aspects. For instance, a study indicated weak hospital readiness for confronting COVID-19 in Sistan and Baluchestan, with low healthcare services and surge capacity in this province [63]. Interestingly, previous studies have shown that Alborz is a deprived province regarding healthcare indices [64, 65]. In contrast, provinces like Khorasan Razavi and Qom, boasting better health infrastructures [65], may face challenges due to urbanization and higher connectivity with other provinces [62] along with inequitable distribution of health resources [64], resulting in inappropriate care due to hospital overload. Significant mortality in COVID-19 has been linked to inability of hospitals to provide ICU beds, forced intermittent ventilation, and intensive care [66]. Additionally, environmental properties [58] and travel patterns [67] contribute to the transmission of COVID-19. Previous representative national studies, such as the STEPwise approach to NCD risk factor surveillance (STEPS) in Iran [68, 69], have highlighted inequalities at both the province and district levels in hypertension and diabetes prevalence [70, 71], their cascade of care [72, 73], and NCDs risk factors [74, 75]. Consequently, further research is necessary to comprehend the causes of these provincial differences, ultimately informing evidence-based policymaking for future health problems.

The COVID-19 pandemic, imposed a significant health and economic burden, challenged and highlighted the efficacy of health systems in managing disease surges. This study reveals disparities in mortality and hospitalization duration among provinces. Further investigation into age and sex structure changes, along with utilizing studies and claims data, informs policies for better hospital distribution, enhancing resilience to future health challenges. The pandemic emphasized the importance of predicting disease hospitalization duration for efficient health policy-making [54], recommending the use of predictive and machine learning models for improved preparedness and pandemic control [76, 77]. Increasing registry usage, like IHIO hospitalization data, warrants enhancements for comprehensive analysis by incorporating variables such as clinical symptoms and underlying diseases when designing the registries, benefiting insurance companies and policymakers in detecting hospital malfunctions and improving overall functionality.

The current study had some limitations. The data source used for analysis and investigation of the study aims was the main limitation as a registry of patients during COVID-19 could have a degree of missing values and not define some important variables in the database, such as clinical symptoms of patients. There is also the probability of wrong detection of diseases at the first place, leading to wrong ICD-10 recording in the database. Another limitation arose from the lack of data on patients covered by different health insurance companies, representing a substantial portion of Iran’s residents—approximately one-third to half. This could potentially impact the generalization of our study results, emphasizing the need for further research into the more commonly employed types of health insurance organizations and companies. Furthermore, it’s important to note that not all hospitals in Iran have contracts with IHIO. On the other hand, this study had several strengths, including having real-world data on hospitalized COVID-19 patients, free of self-reported biases like recall bias. To our knowledge, this study has the largest sample size, encompassing all provinces of Iran, among studies related to this subject in the country. Also, the inclusion of patients and cleaning the data according to defining cases based on hospitalization intervals and aggregation of short interval data added to the strength of analysis conducted in this study. The methodology of the current investigation could be a successful sample for analysis of the claims data in developing countries like Iran with evolving registry systems and healthcare database development efforts.

## Conclusions

Utilizing hospitalization registry data from one of Iran’s most widely used public health insurance programs, this study examined hospitalization durations, mortality rates, and associated factors within a vast nationwide population of hospitalized patients. Our findings indicate that increasing age and admission to the ICU were associated with both longer hospital stays and higher mortality rates, while males and foreigners faced an elevated risk of mortality. Additionally, our study unveiled variations among provinces in both outcomes after adjusting for various variables. With the ongoing advancements in registry systems and claims data, improving these systems could transform them into valuable sources of information for policymakers and health strategists. By investigating these data alongside other studies on population structure and NCDs, we can evaluate the performance and effectiveness of healthcare systems at both provincial and hospital levels. This evidence-based approach facilitates the optimal allocation of resources, enhancing the resilience of the health system in the face of future challenges.

## Supporting information

S1 Fig Distribution of cases among age groups in each province in this study. (TIF)

S2 Fig The share of age groups in dead patients hospitalized with COVID-19 during the study period. (TIF)

S3 Fig Mean number of hospitalization duration days based on sex, and age groups. A) Overall hospitalization duration days, B) ICU duration days.
(TIF)

S4 Fig Mean number of hospitalization duration days based on specialists managing the hospitalized patients. A) Overall hospitalization duration days, B) ICU duration days.
(TIF)

S1 Table Subnational distribution of number of defined cases in provinces bases on crude number of cases, and number of cases pers 100 000 insured population. (DOCX)

S2 Table Case fatality and recovery rates in different provinces of Iran among included cases. (DOCX)

S3 Table Association of mortality and median hospitalization period in different provinces of Iran in this study. (DOCX)

S4 Table Overall and ICU hospitalization period mean based on admission type, insurance type and outcome. (DOCX)

S1 File Comprehensive details of alterations to variables after defining the cases. (DOCX)
