# Supplementary figures and images for: Patterns of case fatality and hospitalization duration among nearly 1 million hospitalized COVID-19 patients covered by Iran Health Insurance Organization (IHIO) over two years of pandemic: An analysis of associated factors
Source: PLoS One. 2024 Feb 23;19(2):e0298604. doi: 10.1371/journal.pone.0298604 (PMC10889889; doi:10.1371/journal.pone.0298604)

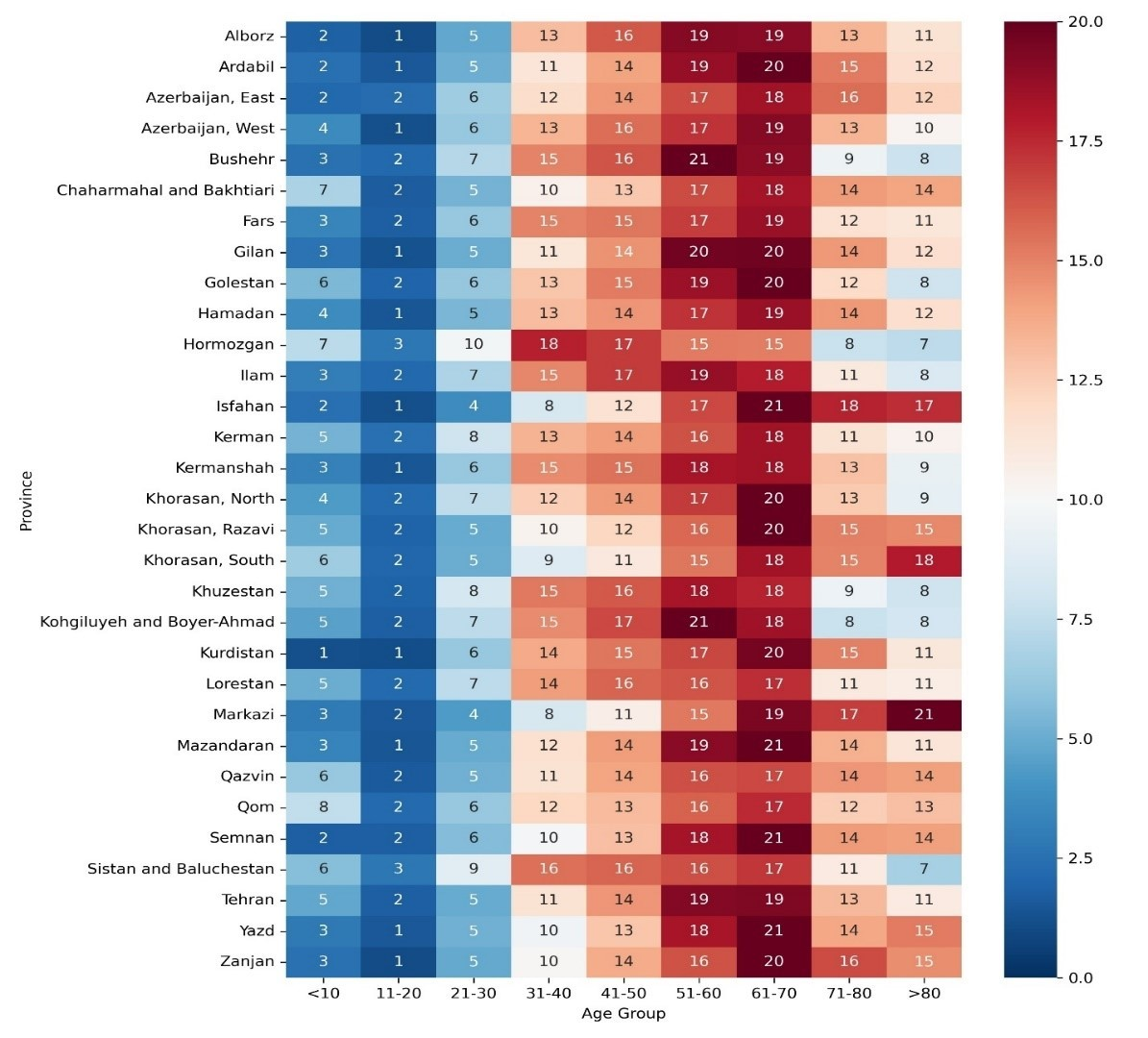

Supplement: S1 Fig — (TIF) [file pone.0298604.s001.tif]

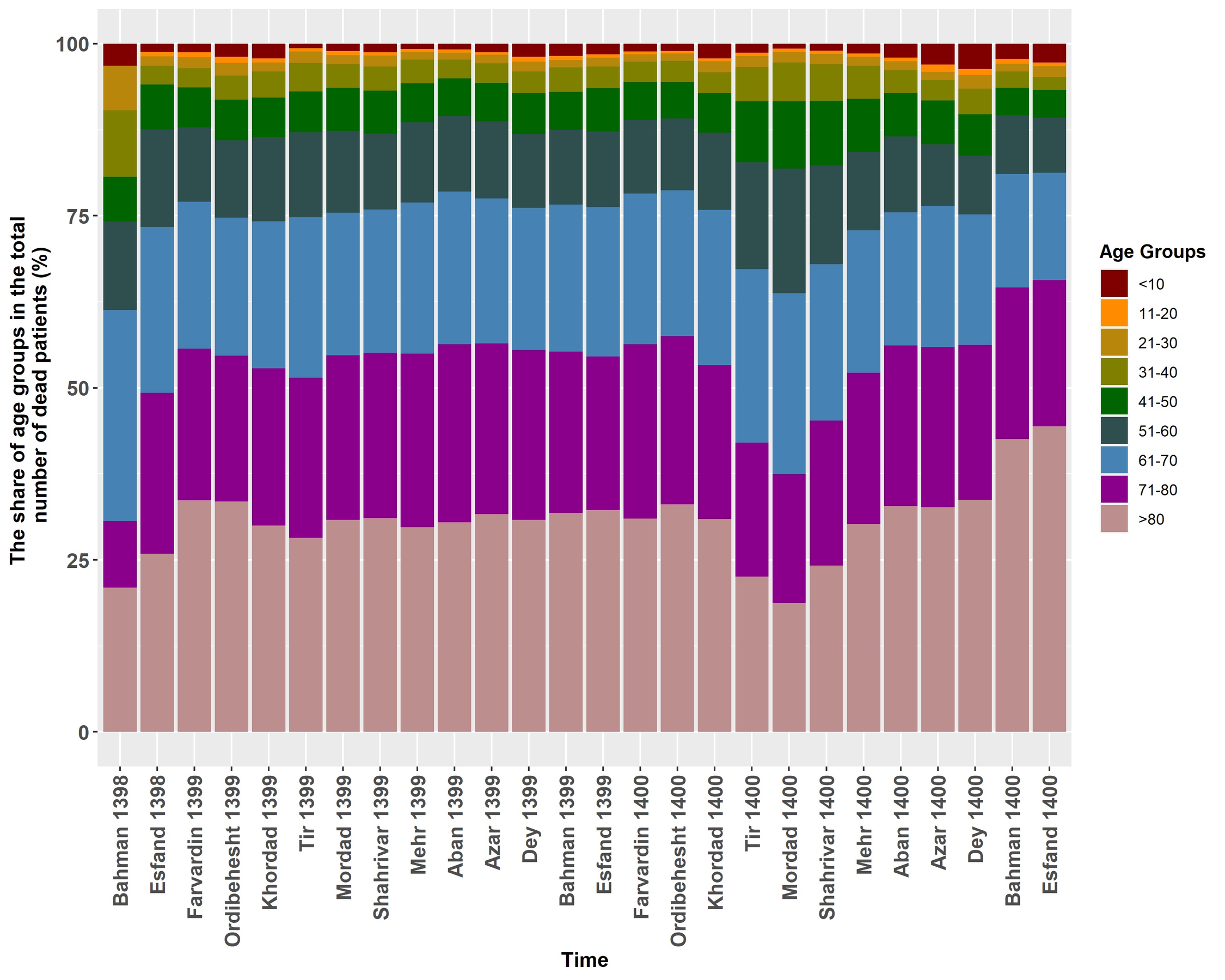

Supplement: S2 Fig — (TIF) [file pone.0298604.s002.tif]

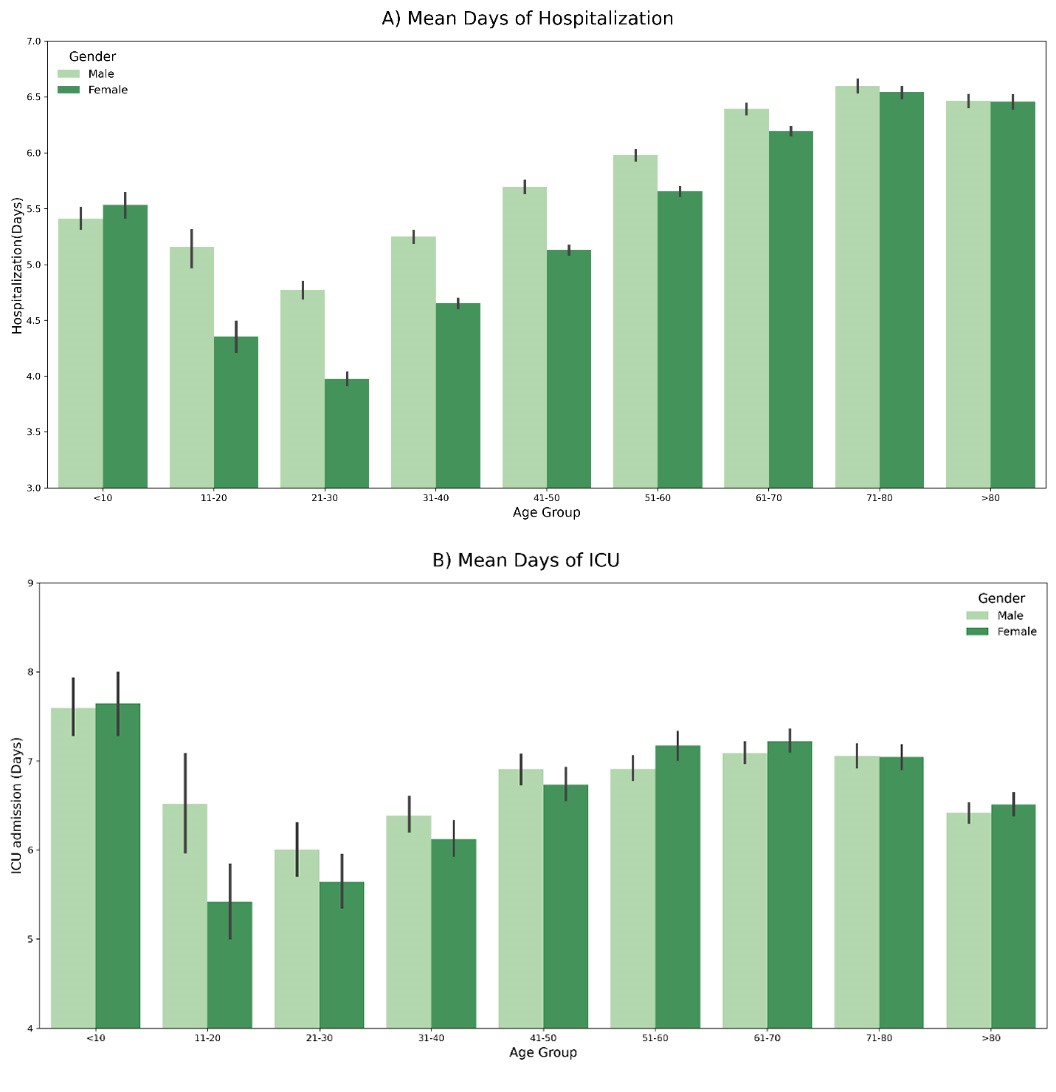

Supplement: S3 Fig — A) Overall hospitalization duration days, B) ICU duration days. (TIF) [file pone.0298604.s003.tif]

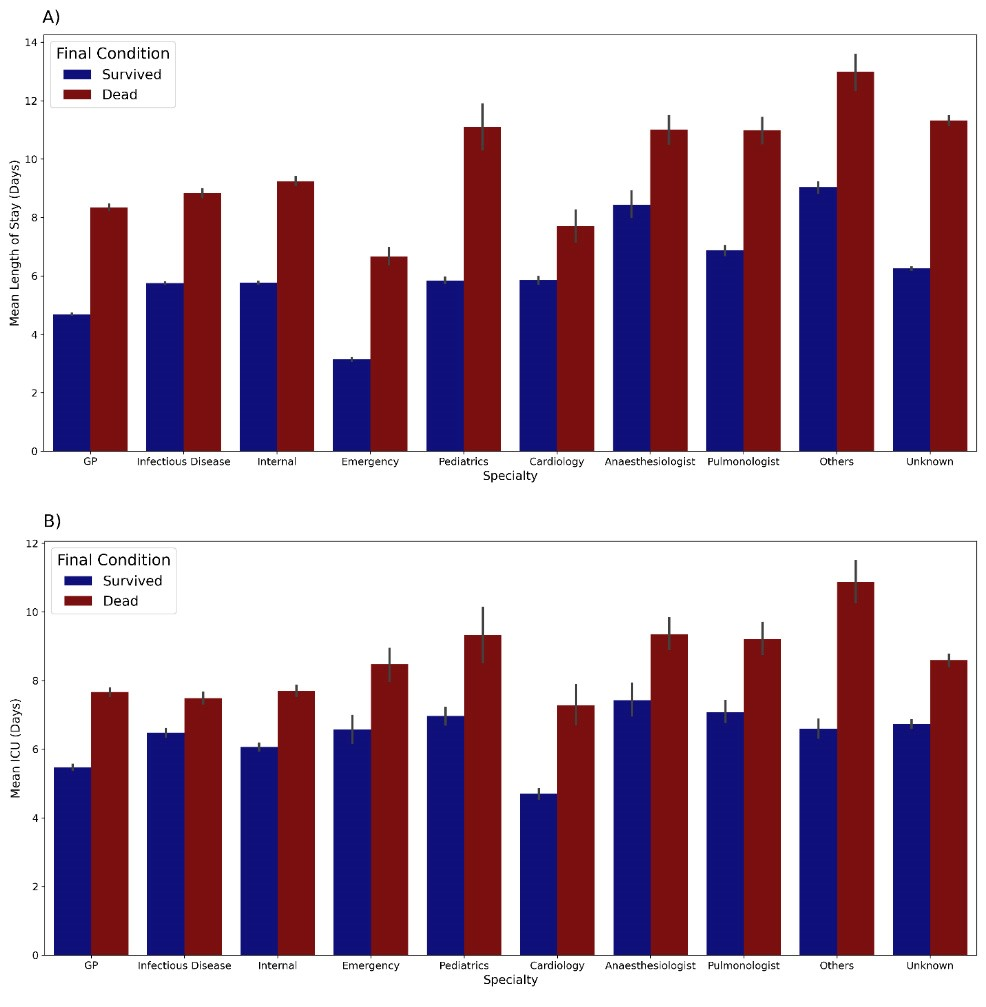

Supplement: S4 Fig — A) Overall hospitalization duration days, B) ICU duration days. (TIF) [file pone.0298604.s004.tif]
