# Supplementary material for: Patterns of case fatality and hospitalization duration among nearly 1 million hospitalized COVID-19 patients covered by Iran Health Insurance Organization (IHIO) over two years of pandemic: An analysis of associated factors
Source: PLoS One. 2024 Feb 23;19(2):e0298604. doi: 10.1371/journal.pone.0298604 (PMC10889889; doi:10.1371/journal.pone.0298604)
Supplement: S1 Table — (DOCX) [file pone.0298604.s005.docx]

**S1 Table.** Subnational distribution of number of defined cases in provinces bases on crude number of cases, and number of cases pers 100 000 insured population.

| **Province** | **Number of cases** | **Population with insurance** | **Standardized number of cases** |
| --- | --- | --- | --- |
| Alborz | 17520 | 748,179 | 2342 |
| Ardabil | 18569 | 856,438 | 2168 |
| Azerbaijan, East | 57369 | 2,228,021 | 2575 |
| Azerbaijan, West | 49730 | 2,369,369 | 2099 |
| Bushehr | 8427 | 458,431 | 1838 |
| Chaharmahal and Bakhtiari | 13208 | 631,670 | 2091 |
| Fars | 56427 | 3,032,105 | 1861 |
| Gilan | 25731 | 1,438,465 | 1789 |
| Golestan | 26150 | 1,277,696 | 2047 |
| Hamadan | 34505 | 1,189,197 | 2902 |
| Hormozgan | 23422 | 1,113,267 | 2104 |
| Ilam | 12945 | 392,762 | 3296 |
| Isfahan | 54277 | 1,749,655 | 3102 |
| Kerman | 45144 | 1,728,613 | 2612 |
| Kermanshah | 27819 | 1,307,856 | 2127 |
| Khorasan, North | 18450 | 706,026 | 2613 |
| Khorasan, Razavi | 71558 | 4,209,027 | 1700 |
| Khorasan, South | 14536 | 513,666 | 2830 |
| Khuzestan | 51772 | 2,570,575 | 2014 |
| Kohgiluyeh and Boyer-Ahmad | 12564 | 514,352 | 2443 |
| Kurdistan | 22469 | 1,163,583 | 1931 |
| Lorestan | 36503 | 1,244,418 | 2933 |
| Markazi | 13626 | 608,727 | 2238 |
| Mazandaran | 49620 | 1,630,546 | 3043 |
| Qazvin | 11641 | 559,939 | 2079 |
| Qom | 16862 | 574,849 | 2933 |
| Semnan | 9010 | 237,842 | 3788 |
| Sistan and Baluchestan | 15745 | 2,472,657 | 637 |
| Tehran | 70458 | 3,808,235 | 1850 |
| Yazd | 13143 | 318,380 | 4128 |
| Zanjan | 14699 | 623,791 | 2356 |
