# Supplementary material for: Patterns of case fatality and hospitalization duration among nearly 1 million hospitalized COVID-19 patients covered by Iran Health Insurance Organization (IHIO) over two years of pandemic: An analysis of associated factors
Source: PLoS One. 2024 Feb 23;19(2):e0298604. doi: 10.1371/journal.pone.0298604 (PMC10889889; doi:10.1371/journal.pone.0298604)
Supplement: S2 Table — (DOCX) [file pone.0298604.s006.docx]

**S2 Table 2.** Case fatality and recovery rates in different provinces of Iran among included cases.

| **Province** | **Recovered**  **No. (%) (95% Confidence Interval)** | **Dead**  **No. (%) (95% Confidence Interval)** | **Total**  **No. (%) (95% Confidence Interval)** |
| --- | --- | --- | --- |
| Markazi | 12167 (89.29%)(88.77-89.81) | 1459 (10.71%)(10.19-11.23) | 13626 (1.49%)(1.47-1.52) |
| Gilan | 22958 (89.22%)(88.84-89.6) | 2773 (10.78%)(10.4-11.16) | 25731 (2.82%)(2.78-2.85) |
| Mazandaran | 45240 (91.17%)(90.92-91.42) | 4380 (8.83%)(8.58-9.08) | 49620 (5.43%)(5.38-5.48) |
| Azerbaijan, East | 51833 (90.35%)(90.11-90.59) | 5536 (9.65%)(9.41-9.89) | 57369 (6.28%)(6.23-6.33) |
| Azerbaijan, West | 44761 (90.01%)(89.74-90.27) | 4969 (9.99%)(9.73-10.26) | 49730 (5.44%)(5.4-5.49) |
| Kermanshah | 25453 (91.5%)(91.17-91.82) | 2366 (8.5%)(8.18-8.83) | 27819 (3.04%)(3.01-3.08) |
| Khuzestan | 46891 (90.57%)(90.32-90.82) | 4881 (9.43%)(9.18-9.68) | 51772 (5.66%)(5.62-5.71) |
| Fars | 51196 (90.73%)(90.49-90.97) | 5231 (9.27%)(9.03-9.51) | 56427 (6.17%)(6.12-6.22) |
| Kerman | 41300 (91.49%)(91.23-91.74) | 3844 (8.51%)(8.26-8.77) | 45144 (4.94%)(4.9-4.98) |
| Khorasan, Razavi | 60027 (83.89%)(83.62-84.16) | 11531 (16.11%)(15.84-16.38) | 71558 (7.83%)(7.77-7.89) |
| Isfahan | 47584 (87.67%)(87.39-87.95) | 6693 (12.33%)(12.05-12.61) | 54277 (5.94%)(5.89-5.99) |
| Sistan and Baluchestan | 13793 (87.6%)(87.09-88.12) | 1952 (12.4%)(11.88-12.91) | 15745 (1.72%)(1.7-1.75) |
| Kurdistan | 20671 (92.0%)(91.64-92.35) | 1798 (8.0%)(7.65-8.36) | 22469 (2.46%)(2.43-2.49) |
| Hamadan | 32103 (93.04%)(92.77-93.31) | 2402 (6.96%)(6.69-7.23) | 34505 (3.78%)(3.74-3.81) |
| Chaharmahal and Bakhtiari | 12033 (91.1%)(90.62-91.59) | 1175 (8.9%)(8.41-9.38) | 13208 (1.45%)(1.42-1.47) |
| Lorestan | 34061 (93.31%)(93.05-93.57) | 2442 (6.69%)(6.43-6.95) | 36503 (3.99%)(3.95-4.03) |
| Ilam | 12214 (94.35%)(93.96-94.75) | 731 (5.65%)(5.25-6.04) | 12945 (1.42%)(1.39-1.44) |
| Kohgiluyeh and Boyer-Ahmad | 12089 (96.22%)(95.89-96.55) | 475 (3.78%)(3.45-4.11) | 12564 (1.37%)(1.35-1.4) |
| Bushehr | 7707 (91.46%)(90.86-92.05) | 720 (8.54%)(7.95-9.14) | 8427 (0.92%)(0.9-0.94) |
| Zanjan | 13231 (90.01%)(89.53-90.5) | 1468 (9.99%)(9.5-10.47) | 14699 (1.61%)(1.58-1.63) |
| Semnan | 8057 (89.42%)(88.79-90.06) | 953 (10.58%)(9.94-11.21) | 9010 (0.99%)(0.97-1.01) |
| Yazd | 12088 (91.97%)(91.51-92.44) | 1055 (8.03%)(7.56-8.49) | 13143 (1.44%)(1.41-1.46) |
| Hormozgan | 21899 (93.5%)(93.18-93.81) | 1523 (6.5%)(6.19-6.82) | 23422 (2.56%)(2.53-2.6) |
| Tehran | 61232 (86.91%)(86.66-87.15) | 9226 (13.09%)(12.85-13.34) | 70458 (7.71%)(7.65-7.76) |
| Ardabil | 16702 (89.95%)(89.51-90.38) | 1867 (10.05%)(9.62-10.49) | 18569 (2.03%)(2.0-2.06) |
| Qom | 14409 (85.45%)(84.92-85.98) | 2453 (14.55%)(14.02-15.08) | 16862 (1.85%)(1.82-1.87) |
| Qazvin | 10148 (87.17%)(86.57-87.78) | 1493 (12.83%)(12.22-13.43) | 11641 (1.27%)(1.25-1.3) |
| Golestan | 22860 (87.42%)(87.02-87.82) | 3290 (12.58%)(12.18-12.98) | 26150 (2.86%)(2.83-2.9) |
| Khorasan, North | 16761 (90.85%)(90.43-91.26) | 1689 (9.15%)(8.74-9.57) | 18450 (2.02%)(1.99-2.05) |
| Khorasan, South | 13337 (91.75%)(91.3-92.2) | 1199 (8.25%)(7.8-8.7) | 14536 (1.59%)(1.56-1.62) |
| Alborz | 14830 (84.65%)(84.11-85.18) | 2690 (15.35%)(14.82-15.89) | 17520 (1.92%)(1.89-1.95) |
| Total | 819635 (89.69%)(89.62-89.75) | 94264 (10.31%)(10.25-10.38) | 913899 (100.0%)(100.0-100.0) |
