# Supplementary material for: Patterns of case fatality and hospitalization duration among nearly 1 million hospitalized COVID-19 patients covered by Iran Health Insurance Organization (IHIO) over two years of pandemic: An analysis of associated factors
Source: PLoS One. 2024 Feb 23;19(2):e0298604. doi: 10.1371/journal.pone.0298604 (PMC10889889; doi:10.1371/journal.pone.0298604)
Supplement: S3 Table — (DOCX) [file pone.0298604.s007.docx]

**S3 Table.** Association of mortality and median hospitalization period in different provinces of Iran in this study.

|  | **Mortality** | | **hospitalization period** | |
| --- | --- | --- | --- | --- |
| Province | **Total cases**  **Adjusted Odds Ratio (aOR) (95% CI)** | **ICU admitted cases**  **Adjusted Odds Ratio (aOR) (95% CI)** | **Overall hospitalization days**  **Adjusted Median Ratio (aMR) (95% CI)** | **ICU hospitalization days**  **Adjusted Median Ratio (aMR) (95% CI)** |
| Markazi | 0.59 (0.55-0.63) | 0.6 (0.56-0.64) | 0.96 (0.94-0.97) | 0.88 (0.87-0.89) |
| Gilan | 0.84 (0.8-0.88) | 0.86 (0.82-0.9) | 1.03 (1.02-1.04) | 0.88 (0.88-0.89) |
| Mazandaran | 0.61 (0.59-0.64) | 0.67 (0.64-0.7) | 0.92 (0.92-0.93) | 0.93 (0.93-0.94) |
| Azerbaijan, East | 0.77 (0.75-0.81) | 0.78 (0.75-0.81) | 0.89 (0.88-0.89) | 0.93 (0.93-0.94) |
| Azerbaijan, West | 0.75 (0.72-0.78) | 0.75 (0.72-0.78) | 0.95 (0.94-0.96) | 0.89 (0.89-0.9) |
| Kermanshah | 0.68 (0.65-0.72) | 0.71 (0.67-0.75) | 0.84 (0.83-0.85) | 0.92 (0.91-0.92) |
| Khuzestan | 0.99 (0.95-1.03) | 1.07 (1.03-1.11) | 1.04 (1.03-1.05) | 0.95 (0.95-0.96) |
| Fars | 0.78 (0.75-0.81) | 0.82 (0.79-0.86) | 0.98 (0.98-0.99) | 0.89 (0.89-0.9) |
| Kerman | 0.75 (0.72-0.78) | 0.78 (0.74-0.81) | 0.96 (0.95-0.97) | 0.89 (0.88-0.89) |
| Khorasan, Razavi | 1.24 (1.2-1.28) | 1.25 (1.21-1.29) | 1.02 (1.01-1.03) | 0.93 (0.92-0.93) |
| Isfahan | 0.72 (0.69-0.74) | 0.73 (0.7-0.76) | 1.0 (0.99-1.0) | 0.88 (0.88-0.89) |
| Sistan and Baluchestan | 1.4 (1.32-1.48) | 1.43 (1.35-1.52) | 0.94 (0.93-0.95) | 0.91 (0.9-0.91) |
| Kurdistan | 0.52 (0.49-0.55) | 0.52 (0.49-0.55) | 0.79 (0.79-0.8) | 0.85 (0.85-0.86) |
| Hamadan | 0.51 (0.49-0.54) | 0.53 (0.5-0.56) | 1.12 (1.11-1.13) | 0.9 (0.89-0.91) |
| Chaharmahal and Bakhtiari | 0.64 (0.6-0.68) | 0.66 (0.61-0.71) | 1.03 (1.01-1.04) | 0.88 (0.87-0.88) |
| Lorestan | 0.56 (0.53-0.59) | 0.58 (0.55-0.61) | 0.92 (0.91-0.93) | 0.89 (0.89-0.9) |
| Ilam | 0.51 (0.47-0.56) | 0.53 (0.49-0.57) | 0.87 (0.86-0.88) | 0.92 (0.91-0.93) |
| Kohgiluyeh and Boyer-Ahmad | 0.3 (0.28-0.34) | 0.33 (0.3-0.36) | 0.94 (0.92-0.95) | 0.84 (0.83-0.85) |
| Bushehr | 0.73 (0.68-0.8) | 0.73 (0.67-0.8) | 0.82 (0.81-0.83) | 0.87 (0.87-0.88) |
| Zanjan | 0.63 (0.6-0.67) | 0.65 (0.61-0.69) | 1.02 (1.0-1.03) | 0.88 (0.88-0.89) |
| Semnan | 0.76 (0.7-0.82) | 0.76 (0.7-0.82) | 0.9 (0.89-0.92) | 0.95 (0.94-0.96) |
| Yazd | 0.5 (0.47-0.53) | 0.51 (0.47-0.54) | 0.87 (0.86-0.88) | 0.87 (0.86-0.88) |
| Hormozgan | 0.7 (0.66-0.74) | 0.7 (0.66-0.75) | 0.81 (0.8-0.82) | 0.9 (0.89-0.91) |
| Tehran | 0.67 (0.63-0.7) | 0.71 (0.67-0.75) | 1.05 (1.04-1.06) | 0.88 (0.87-0.88) |
| Ardabil | 1.13 (1.07-1.19) | 1.17 (1.1-1.23) | 0.98 (0.97-1.0) | 0.91 (0.9-0.92) |
| Qom | 0.88 (0.82-0.93) | 0.88 (0.83-0.94) | 1.0 (0.98-1.02) | 0.92 (0.92-0.93) |
| Qazvin | 1.06 (1.01-1.11) | 1.19 (1.14-1.25) | 0.96 (0.95-0.97) | 0.92 (0.92-0.93) |
| Golestan | 0.74 (0.7-0.79) | 0.76 (0.72-0.81) | 0.88 (0.87-0.89) | 0.89 (0.88-0.9) |
| Khorasan, North | 0.51 (0.48-0.54) | 0.52 (0.49-0.56) | 0.73 (0.72-0.74) | 0.86 (0.85-0.87) |
| Khorasan, South | 1.28 (1.22-1.35) | 1.3 (1.23-1.37) | 1.1 (1.08-1.11) | 0.97 (0.96-0.98) |
| Alborz | 0.59 (0.55-0.63) | 0.6 (0.56-0.64) | 0.96 (0.94-0.97) | 0.88 (0.87-0.89) |

* Adjustments were made with sex, age, insurance fund, admission type, the month of admission, and specialty of the physician

**Reference Province: Tehran

***95% CI: 95% Confidence Interval
