# Supplementary material for: Patterns of case fatality and hospitalization duration among nearly 1 million hospitalized COVID-19 patients covered by Iran Health Insurance Organization (IHIO) over two years of pandemic: An analysis of associated factors
Source: PLoS One. 2024 Feb 23;19(2):e0298604. doi: 10.1371/journal.pone.0298604 (PMC10889889; doi:10.1371/journal.pone.0298604)
Supplement: S4 Table — (DOCX) [file pone.0298604.s008.docx]

**S4 Table.** Overall and ICU hospitalization period mean based on admission type, insurance type and outcome.

| **Variables** | **Overall hospitalization day mean (95% CI)** | **ICU hospitalization day mean (95 % CI)** |
| --- | --- | --- |
| **Admission Type** |  |  |
| Ward | 6.51 (6.50-6.53) | 6.85 (6.81-6.89) |
| Emergency department | 2.37 (2.35-2.38) | 1.50 (0.11-2.89) |
| **Insurance type** |  |  |
| Rural | 5.53 (5.51-5.55) | 6.48 (6.42-6.55) |
| Civil Servants | 5.90 (5.88-5.93) | 7.22 (7.14-7.30) |
| Other Social Strata | 6.31 (6.27-6.35) | 6.81 (6.70-6.91) |
| Foreign Citizens | 6.04 (5.85-6.23) | 7.31 (6.89-7.74) |
| Iranian | 6.78 (6.70-6.86) | 7.35 (7.17-7.52) |
| Universal Health Insurance | 5.60 (5.57-5.63) | 6.82 (6.74-6.91) |
| Unknown | 9.49 (9.08-9.90) | 9.63 (8.08-10.65) |
| **Outcome** |  |  |
| Recovery | 9.26 (9.20-9.32) | 8.00 (7.93-8.07) |
| Death | 5.38 (5.37-5.39) | 6.06 (6.02-6.11) |
| 95% CI: 95% Confidence Interval | | |
