# Supplementary material for: Patterns of case fatality and hospitalization duration among nearly 1 million hospitalized COVID-19 patients covered by Iran Health Insurance Organization (IHIO) over two years of pandemic: An analysis of associated factors
Source: PLoS One. 2024 Feb 23;19(2):e0298604. doi: 10.1371/journal.pone.0298604 (PMC10889889; doi:10.1371/journal.pone.0298604)
Supplement: S1 File — (DOCX) [file pone.0298604.s009.docx]

**Supplementary Appendix**

**Materials and methods:**

***Data preparation***

After creating the table of newly defined cases as explained in the main text, there was a need for standardizing various variable states for each case, whenever possible. Consider, for example, an aggregated admission that was originally derived from three separate admissions occurring within a 30 days interval. The row corresponding to this admission included information for three ages, three physician specialists, three types of admissions, etc. To incorporate this admission into our analyses, we had to select one age, one physician specialist, one type of admission, and so forth for this entry. Otherwise, the admission could not be used as a variable in our description and analysis. Below, we provide an explanation of how each variable was aggregated:

**Age:** If all ages within the aggregated admissions were the same, that specific age was considered. However, when ages varied, the age with the highest frequency was selected as the final age. If more than one age had the highest frequency, the mean of those two ages was considered. Ultimately, admissions with patient ages less than 1 year or over 105 years were excluded from the study.

**Gender:** In cases where more than two genders were recorded for a patient within an aggregated admission, the gender with the highest frequency was considered. If the patient's gender remained unspecified or marked as 'missing value', the case was excluded from the study.

**Length of Hospital Stay (Total, Ward, ICU):** The days spent in different types of hospitalization were summed up for each admission. In the original data, patients with admission and discharge dates on the same day were assigned zero hospitalization days. In the aggregated data, zero hospitalization days were treated as one full hospitalization day, and they were then added to the other aggregated hospitalization days. Admissions that lasted over 90 days were excluded from the study.

**Insurance Fund Type, Province, Physician Specialty:** If all instances within aggregated admissions had the same values for these variables, those values were retained. Otherwise, no aggregation was performed for these variables. In analyses and descriptions requiring the use of these variables, they were placed under the 'unspecified' category for insurance funds and physician specialties, while for provinces, they were not used.

**COVID-19 Diagnosis Code:** If only one U07.1 code existed in an aggregated admission, the diagnosis code was considered as U07.1; otherwise, it was marked as U07.2.

**Admission Type:** If only one "ward admission" existed in an aggregated admission, the admission type was considered as "ward admission." Otherwise, it was considered as "emergency department admission".

**Hospitalization Outcome:** Existing of only one "death" outcome in an aggregated admission, would make the outcome as “death”; otherwise, it was considered as "recovery".
